# Announcement

**DOI:** 10.1155/S1463924698000078

**Published:** 1998


					Journal of Automatic Chemistry, Vol. 20, No. 2 (March-April 1998) pp. 67
Announcement
ISLAR '97 recognizes pioneers in laboratory automation and
robotics
The fifteenth International Symposium on Laboratory
Automation and Robotics, held at the Boston Park Plaza
Hotel during 19-20 October 1997 recognized 11 scientists
for their roles in developing new applications and
techniques, designing unique solutions to technical prob-
lems, and implementing laboratory robotics in their
organizations. Nominated by colleagues in their com-
panies or industry, these scientists were selected for this
recognition by a committee of previous award winners.
The scientists included John Bahia, Wyeth-Ayerst,
Princeton, NJ; Allen Carman, US FDA, New Orleans,
LA; Scott Jennings, Bristol-Meyers Squibb, New Bruns-
wick, NJ; Kirk J. Leister, Bristol-Myers Squibb,
Syracuse, NY; Dinesh S. Thakur, Bristol-Myers Squibb,
Syracuse, NY; Lars W. Linquist, WMX Technology
Center, Geneva, IL; Thomas L. Lloyd, DuPont Merck,
Newark, DE; Martin Phillippi, Clorox Company,
Pleasanton, CA; Danh Nguygen, Clorox Company,
Pleasanton, CA; Michael E. Rusak, Air Products and
Chemicals, Allentown, PA; Michael L. Rutherford, Eli
Lilly and Company, Indianapolis, IN.
Islar '97 attracted more than 600 scientists and managers
from 34 states and 15 countries for three days of
presentations on laboratory automation and robotics.
The premier technical symposium in this field featured
a programme with over 125 papers and posters, six
discussion groups, several workshops and six short
courses. Session topics included:
High throughput screening
Data management/data handling
Re-engineering the laboratory
Managing laboratory automation
Pharmateutical analysis
Bioanalytical assays
Automated synthesis
Compound purification, distribution and analysis in
drug discovery
Laboratory workstations
Chemical/agriculture chemical analysis
Advanced topics.
For more information, contact Sharon Correia, ISLAR Program
Committee, 68 Elm Street, Hopkinton, MA 01748, Tel. 508 497-
6403, Fax. 508 435-3439, email." islar@islar.com, website
http www.islar.com
0142-0453/98 $12.00 (C) 1998 Taylor & Francis I,td
67

				

